# Acute Stress Reduces Wound-Induced Activation of Microbicidal Potential of Ex Vivo Isolated Human Monocyte-Derived Macrophages

**DOI:** 10.1371/journal.pone.0055875

**Published:** 2013-02-08

**Authors:** Ulrike Kuebler, Petra H. Wirtz, Miho Sakai, Andreas Stemmer, Ulrike Ehlert

**Affiliations:** 1 Department of Clinical Psychology and Psychotherapy, University of Zurich, Zurich, Switzerland; 2 Biological and Health Psychology, University of Bern, Bern, Switzerland; 3 Nanotechnology Group, Swiss Federal Institute of Technology Zurich, Zurich, Switzerland; Charite Universitätsmedizin, Germany

## Abstract

**Background:**

Psychological stress delays wound healing but the precise underlying mechanisms are unclear. Macrophages play an important role in wound healing, in particular by killing microbes. We hypothesized that (a) acute psychological stress reduces wound-induced activation of microbicidal potential of human monocyte-derived macrophages (HMDM), and (b) that these reductions are modulated by stress hormone release.

**Methods:**

Fourty-one healthy men (mean age 35±13 years) were randomly assigned to either a stress or stress-control group. While the stress group underwent a standardized short-term psychological stress task after catheter-induced wound infliction, stress-controls did not. Catheter insertion was controlled. Assessing the microbicidal potential, we investigated PMA-activated superoxide anion production by HMDM immediately before and 1, 10 and 60 min after stress/rest. Moreover, plasma norepinephrine and epinephrine and salivary cortisol were repeatedly measured. In subsequent *in vitro* studies, whole blood was incubated with norepinephrine in the presence or absence of phentolamine (norepinephrine blocker) before assessing HMDM microbicidal potential.

**Results:**

Compared with stress-controls, HMDM of the stressed subjects displayed decreased superoxide anion-responses after stress (*p*’s <.05). Higher plasma norepinephrine levels statistically mediated lower amounts of superoxide anion-responses (indirect effect 95% CI: 4.14–44.72). Norepinephrine-treated HMDM showed reduced superoxide anion-production (*p*<.001). This effect was blocked by prior incubation with phentolamine.

**Conclusions:**

Our results suggest that acute psychological stress reduces wound-induced activation of microbicidal potential of HMDM and that this reduction is mediated by norepinephrine. This might have implications for stress-induced impairment in wound healing.

## Introduction

Animal and human studies provide converging evidence that acute as well as chronic psychological stress substantially delays skin wound healing [1]. Although current findings suggest that the stress-induced delay of wound healing is associated with a suppressed immune reaction in early phases of wound healing, the precise underlying mechanisms of this stress effect remain unclear [2].

Macrophages are tissue-resident immune cells that are differentiated from circulating blood monocytes. When activated, macrophages acquire competence to perform a variety of critical immunological functions, including the killing of microbes (i.e. microbicidal activity) and tumor cells, regulation of lymphocytes and inflammation, or remodeling and repair of tissue [3]–[5]. In particular, macrophage microbicidal activity plays a significant role in early phases of inflammation such as the first phases of skin wound healing [6]. Macrophage activation in terms of microbicidal activity can be investigated in pro-inflammatory M1 macrophages, a phenotype developed following activation with inflammatory cytokines (e.g. IFN-γ, TNF-α) and bacterial products (e.g. LPS) [3], [7]. M1 macrophages are characterized by enhanced microbicidal activity [8], [9], which is mainly mediated by increased secretion of microbe killing highly oxidizing agents, the so-called reactive oxygen species (ROS; e.g. superoxide anion, hydrogen peroxide) and reactive nitrogen species (RNS; e.g. nitric oxide) [3], [10]. Moreover, M1 macrophages secrete high amounts of chemokines and pro-inflammatory cytokines [3].

Given that psychological stress can impair wound healing [1], [11]–[13], and given the role of microbicidal active M1 macrophages in early wound healing phases [6], stress may exert at least parts of its wound healing impairment by inhibiting the microbicidal potential (i.e. ROS production) of these cells. However, the effect of acute psychological stress on ROS-dependent microbicidal potential of *ex vivo* isolated human M1 macrophages has not yet been investigated. To date, inhibitory effects of acute psychological stress on ROS production have only been observed in undifferentiated circulating human blood phagocytes (i.e. neutrophils and monocytes as precursors of macrophages) *in vitro* [14]. Notably, only the investigation of macrophages as tissue cells might allow conclusions regarding microbicidal activity of macrophages at sites of inflammation or wounding. Also, monocytes and macrophages differ considerably in their gene expression profile [15] with the result that findings for monocytes are not necessarily replicated in macrophages and vice versa. Hitherto, studies in rodents investigating the effects of psychological stress on the microbicidal potential of *ex vivo* isolated macrophages reported stress-induced, albeit inconsistent changes in the production of ROS and RNS [16]–[18]. Acute stress may influence M1 microbicidal potential via activation of the two major stress systems, the hypothalamus-pituitary adrenal (HPA) axis and/or the sympathetic nervous system (SNS). Both monocytes and macrophages express receptors for a variety of neuroendocrine products of the HPA axis and the SNS (e.g. receptors for glucocorticoids [GC] and catecholamines [CA] as major stress hormones) [5], [19]–[21]. Moreover, *in vitro* studies exposing non-human macrophages to GC or CA demonstrated predominantly hormone-induced decreases in ROS production [16], [22]. Taken together, mental stress may modulate macrophage microbicidal potential, probably by stress-induced release of GC (as the final products of HPA axis activation) and/or of CA release (resulting from SNS activation).

The purpose of this study was twofold: First, in a sample of healthy men we set out to examine the effects of a potent acute psychological stressor known to induce large stress hormone increases compared to a non-stress control condition on microbicidal potential of M1 macrophages differentiated from pre-activated monocytes (stress study). We inserted a venous catheter which we intended to function as an open wound to pre-activate circulating monocytes as precursors of later M1 macrophages *in vivo.* Psychosocial stress was induced via the widely used Trier Social Stress Test (TSST) [23]. For the investigation of M1 cell microbicidal potential, we performed an WST-1 assay as described elsewhere with minor modifications [24]. The assay principle is based on the reduction of 2-(4-Iodophenyl)-3-(4-nitrophenyl)-5-(2,4-disulfophenyl)-2H-tetrazolium (WST-1) by superoxide anions produced by phorbol 12-myristate 13-acetate (PMA)-activated human monocyte-derived M1 macrophages (HMDM) [25]. Our main hypotheses were that a) catheter-insertion as open wound induction would increase microbicidal potential of HMDM over time, and that b) acute psychological stress would inhibit this wound-induced increase in microbicidal potential. To validate our open wound paradigm, we controlled for catheter insertion by applying a non-open wound blood sampling method (i.e. short-term cannula insertion instead of long-term catheter insertion) in an additional control group.

Second, in order to investigate underlying mechanisms, we tested whether the hypothesized inhibiting effect of stress is statistically mediated by blood norepinephrine (NE), epinephrine (EPI) and/or cortisol levels. Significant mediation effects were further examined in a series of *in vitro* experiments. For this purpose, we set out to incubate whole blood with stress hormones in the presence or absence of the respective stress-hormone-antagonists before performing the WST-1 assay.

## Methods

### Design and Procedure

#### Stress study

Subjects of the stress group and of the stress-control group reported to the laboratory by 10 a.m. and had abstained from extensive physical exercise, alcohol, and caffeinated beverages during the previous 24 h. Subjects were given a calorically standardized breakfast with comparable nutritional composition before an indwelling venous catheter was inserted not only for blood sampling but also to induce an open wound. The following resting period of 165 min was intended to allow *in vivo* stimulation of circulating monocytes by the applied open wound paradigm. Next, subjects of the stress group were exposed to the TSST [23], which comprises a short introduction followed by a 5-min preparation period, a 5-min mock job interview, and a 5-min mental arithmetic task (serial subtraction) in front of an unknown panel of two persons. The TSST has repeatedly been shown to induce significant neuroendocrine stress responses [26]. Subjects of the stress-control group were not exposed to the TSST but were required to stand in the empty TSST room for 10 min in order to control for a possible influence of orthostatic stress. Before and after TSST/rest, subjects remained seated in a quiet room.

Blood samples for WST-1 reduction measurements were obtained immediately before (serving as baseline levels) and 1, 10 and 60 min after TSST/rest cessation. To determine CA levels, blood samples were taken immediately before (baseline) and 1, 10, and 20 min after TSST/rest cessation. For determination of salivary free cortisol levels, samples of saliva were collected immediately before (baseline) and 1, 10, 20, 30, 45, and 60 min after TSST/rest cessation. To determine mean arterial blood pressure (MAP), blood pressure was measured by sphygmomanometry (Omron 773, Omron Healthcare Europe B.V. Hoofdorp, Netherlands) immediately before and 40 min after insertion of the venous catheter. MAP was calculated by the formula (2/3 * mean diastolic BP)+(1/3 mean systolic BP). At the end of the experiment, participants were debriefed and remunerated with 175 Swiss Francs for their participation.

#### Catheter-control: Pilot data

To control for catheter insertion, subjects of the catheter-control group reported to the laboratory for blood collection by short-term cannula venipuncture twice during the same day. The time interval between the two blood samples was intentionally chosen to be comparable to the interval between the first and the last blood sample in the stress study (i.e. baseline assessment and 60 min after stress/rest). Moreover, to account for potential circadian rhythm effects, the first and the last blood sample were drawn at the exact same time of the day as in the stress study (with exception of one subject staring earlier (10 a.m.) and one subject starting later (2 p.m.)). Nine milliliters of blood were drawn from a peripheral vein through disposable cannulas into EDTA-coated tubes (Sarstedt, Numbrecht, Germany) and layered on top of 10 ml Ficoll, and the WST-1 assay was performed as describe below.

#### In vitro experiments

**Figure 1:**
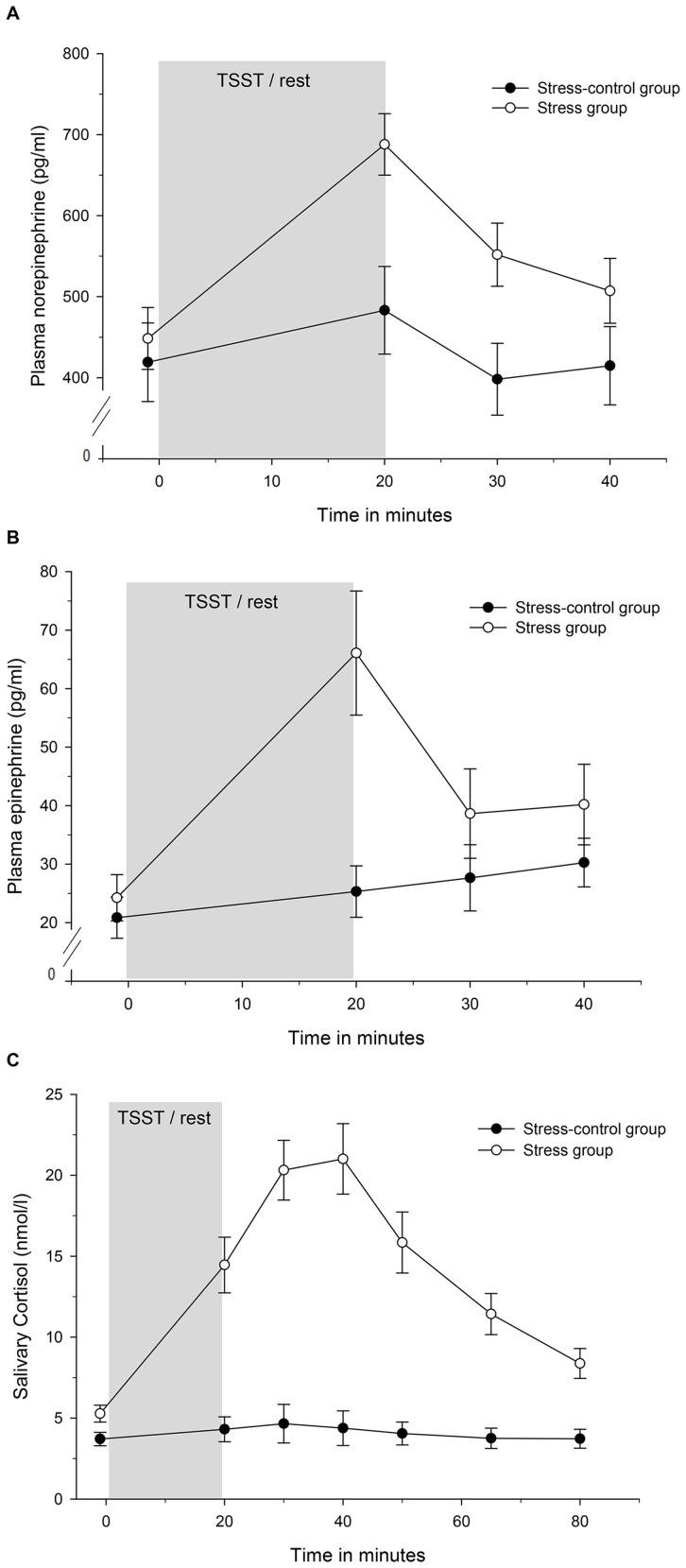
A to C. Course of norepinephrine, epinephrine, and cortisol over time in stress and stress-control group. Values are given as means ± SEM.

In order to assess the effects of physiological post-stress levels of NE on HMDM (cf. Figure 1A), subjects reported to the laboratory for blood collection on a single study day between 11 a.m. and 1 p.m. Nine milliliters of blood were drawn from a peripheral vein through disposable cannulas into EDTA-coated tubes (Sarstedt, Numbrecht, Germany) and incubated with three different concentrations of NE. We added 50 µl of various concentrations of NE to whole blood resulting in a final concentration of 0 (reference sample), 500 (low post-stress levels of NE), and 1000 pg/ml (high post-stress levels of NE) NE. After a 20 min incubation period at 37°C and 5% CO_2_, whole blood was layered on top of 10 ml Ficoll, and the WST-1 assay was performed as described below. For the *in vitro* experiments with the alpha-adrenergic blocker phentolamine used as NE-antagonist, blood samples were incubated with 100 und 1000 pg/ml phenolamine for 10 min before exposure to 500 pg/ml NE for 20 min. Notably, we decided for a stimulation by 500 pg/ml NE as this concentration is close both to the mean NE post stress level (678.95 pg/ml) and to the lowest NE stress increase level (308 pg/ml) that we observed in our stress study. In contrast, a concentration of 1000 pg/ml could be considered too high to be representative for NE stress responses as it is higher than the highest NE stress response (989 pg/ml) that we observed in only one single subject. All *in vitro* experiments were repeated two times and carried out in cells of three female participants, rendering a total of six experiments.

### Participants

#### Stress study

The Ethics Committee of the Canton of Zurich, Switzerland formally approved the research protocol. Subjects were recruited with the aid of the Swiss Red Cross of the Canton of Zurich and through advertisements. All subjects gave written informed consent before participating in the study. We recruited 44 medication-free, healthy Caucasian men between 20 and 50 years of age and obtained complete WST-1 reduction data from 41 of these subjects. Subjects were randomly assigned to either a stress (*n*  = 24) or a stress-control group (*n*  = 20). Three stress-control group subjects had to be excluded due to venous catheter occlusion. Subjects were in good physical and mental health, as confirmed by a telephone interview. Explicit exclusion criteria were: regular strenuous exercise, smoking, alcohol and illicit drug abuse, any heart disease, varicosis or thrombotic diseases, elevated blood sugar and diabetes, elevated cholesterol, liver and renal diseases, chronic obstructive pulmonary disease, allergies and atopic diathesis, rheumatic diseases, and current infectious diseases. If the personal or medication history was not conclusive, the subjects’ primary care physician was contacted for verification.

#### Catheter-control and in vitro experiments

To control for catheter-insertion effects blood samples were taken from eight apparently healthy Caucasian women. For the *in vitro* experiments, blood samples were taken from three apparently healthy Caucasian women, on two different days, rendering a total of six experiments. No exclusion criteria were applied in these eleven blood donators. Notably, catheter-control and *in vitro* experiments were conducted after the stress study for validation purposes based on the results of the stress study.

### Materials and Measurements

#### Reagents and chemicals

We used the following reagents: Ficoll-Paque PLUS (Ficoll; no. 17-1440-02; GE Healthcare; Uppsala, Sweden); 2-(4-Iodophenyl)-3-(4-nitrophenyl)-5-(2,4-disulfophenyl)-2H-tetrazolium (WST-1; no. 150849-52-8, Dojindo Laboratories; Kumamoto, Japan); interferon- γ (IFN-γ; no. PHC4031, Invitrogen; Basel, Switzerland), tumor necrosis factor-α (TNF-α; no. PHC3016, Invitrogen; Basel, Switzerland); Hank’s balanced salt solution without phenol red (HBSS; no. 14025050, Invitrogen; Basel, Switzerland); norepinephrine (Sintetica SA, Mendrisio, Switzerland); phentolamine (regitin®, Novartis Pharma AG, Basel, Switzerland); fetal bovine serum (FBS; no. 10106-169, Invitrogen; Basel, Switzerland); lipopolysaccharide (LPS; no.L6529, Sigma-Aldrich; Buchs, Switzerland); phosphate buffered saline (PBS; no. P5368, Sigma-Aldrich; Buchs, Switzerland); phorbol 12-myristate 13-acetate (PMA; no. P8139, Sigma-Aldrich; Buchs, Switzerland); dimethyl sulfoxide (DMSO; no. D2650, Sigma-Aldrich; Buchs, Switzerland); RPMI-1640 Media with Glutamax (RPMI-1640; no. W9925E, Fisher Scientific; Wohlen, Switzerland).

#### WST-1 assay

The method used to study microbicidal potential of *ex vivo* isolated HMDM is based on that described by Sakai et al. [24]. In brief, 9 ml of blood were collected in EDTA-coated tubes (Sarstedt, Numbrecht, Germany), immediately layered on top of 10 ml Ficoll, and centrifuged for 20 min at 300 g and 20°C. After centrifugation, peripheral blood nuclear cells (PBMCs) were removed from the interface, washed twice in RPMI-1640 medium, counted with a hematologic analyzer (KX-21N; Sysmex Digitana AG), and re-suspended to a concentration of 2.5×10^6^/ml with RPMI-1640 media supplemented with 10% FBS. Then, PBMC suspension aliquots of 1 ml were transferred to 24-well cell culture plates (no. 4609; Semadeni; Ostermundigen, Switzerland). After incubation for 1 h at 37°C and 5% CO_2_, the supernatant was discarded and the plate surface was rinsed five times with 1 ml of warm (25°C) 0.01 M PBS to remove non-adherent PBMC, while monocytes remained adherent to the bottom of the plate.

The adherent monocyte layer was diluted with 1 ml RPMI1640 media supplemented with 10% FBS. Subsequently, we added 5 µl IFN-γ, 2 µl TNF-α, and 0.5 µl LPS resulting in a final concentration of 50 ng/ml IFN-γ, 20 ng/ml TNF-α, and 300 ng/ml LPS to promote differentiation of monocytes into inflammatory macrophages. After incubation for 48 h at 37°C and 5% CO_2_, the supernatant was discarded and the adherent macrophage layer was washed three times with 1 ml of warm (25°C) 0.01 M PBS to remove traces of culture media and non adherent cells.

Next, the resulting macrophage monolayer (obtained as described above) was overlaid with 1 ml HBSS. Subsequently, 2 µl WST-1, 0.5 µl LPS, 5 µl IFN-γ, 2 µl TNF-α, and 0,5 µl PMA were added, resulting in a final concentration of 100 µM WST-1, 300 ng/ml LPS, 50 ng/ml IFN-γ, 20 ng/ml TNF-α, and 50 nM PMA. This was followed by an incubation period of 4 hours at 37°C and 5% CO_2_. Then, the supernatant was removed and used to determine WST-1 reduction by reading the OD with a spectrophotometer (SmartSpec Plus, Bio-Rad Laboratories, Inc.) at 450 nm against water as blank. Higher ODs as obtained in absorbance reading are associated with higher amounts of WST-1 reduced and thus of superoxide anions generated by HMDM [24], [25].

#### Stress hormone assays

For CA assessment, venous blood was drawn in EDTA-coated monovettes (Sarstedt, Numbrecht, Germany), and immediately centrifuged for 10 min at 2000×g and 4°C. Obtained plasma was stored at −80°C until analysis. Plasma CA levels were determined by means of high-pressure liquid chromatography (HPLC) and electrochemical detection after liquid-liquid extraction [27], [28] in the “Laboratory for Stress Monitoring” (Göttingen, Germany). For both NE and EPI, the detection limit was 6 pg/ml and inter- and intra-assay coefficients of variance (CVs) were <5%.

For cortisol, saliva samples were collected in Salivettes (Sarstedt, Sevelen, Switzerland) and stored at −20°C until analysis. Centrifugation of thawed saliva samples was at 3000×g, yielding low-viscosity saliva. Free cortisol concentrations were determined using a commercial chemiluminescence immunoassay (LIA) with high sensitivity of 0.16 ng/ml (IBL Hamburg, Germany). Inter- and intra-assay CVs were <11.5% and 7.7%, respectively.

### Statistical Analysis

Data were analyzed using SPSS Inc. version 17.0 for Windows (Chicago, IL, USA) and presented as mean ± SEM. All tests were two-tailed with the significance level set at *p*≤.05 and the level of borderline significance set at *p*≤.10. Missing data were excluded listwise.

A priori sample size calculations for the stress study were performed using G-power [29]. Based on previous observations [14], we expected a large effect size of 0.40 in terms of stress reactivity of HMDM. Statistical power analyses indicated that the optimal sample size to predict a large effect size of 0.40 in repeated measures analysis of covariance (ANCOVA; with HMDM as repeated factor) with a power of.90 is *n* = 44.

Prior to statistical analyses, all data were tested for normal distribution and homogeneity of variance using Kolmogorov-Smirnov and Levene’s tests. Skewed EPI values were logarithmically transformed and normal distribution was verified.

We determined stress hormone changes by calculating the respective difference between the expected peak stress response minus baseline level. NE and EPI changes (ΔNE; ΔEPI) were calculated as the difference in plasma levels between 1 min post-TSST/rest and baseline. Cortisol stress changes (ΔCORT) refer to changes from baseline to 20 min post-TSST/rest. Body mass index (BMI) was calculated by the formula weight in kg/(height in m)^2^. Due to high levels of inter-individual variations we followed previous research [30] and calculated macrophage microbicidal potential as percentage changes in repeatedly measured WST-1 reduction scores in relation to the respective baseline. Moreover, for repeatedly measured WST-1 reduction, area under the total response curve with respect to ground (AUCg) was calculated with the trapezoid formula [31]. We controlled for age, MAP, and BMI in all WST-1 reduction analyses of the stress study as a priori selected control variables (i.e. covariates in ANCOVAs) based on previous literature suggesting a potential influence on microbicidal potential of HMDM [32]–[34].

Differences between the characteristics of the stress group and the stress-control group were calculated using univariate analysis of variance (ANOVA; Table 2).

**Table 2:** Partial correlations between WST-1 reduction measures and stress hormone change coefficients across all subjects (*n* = 41) after controlling for age, mean arterial blood pressure, and body mass index.

	WST-1 reduction time points after TSST/rest		
	+1 min	+10 min	+60 min
ΔNE (pg/ml)	−.50*	−.34*	−.56**
ΔEPI (pg/ml)	−.21	−.34*	−.10
ΔCORT (nmol/l)	−.21	−.30	−.28

Stress hormone changes (ΔNE, ΔEPI, ΔCORT) refer to the respective difference between the expected stress peak level minus baseline. NE, norepinephrine; EPI, epinephrine; *p<.05, **p<.001.

To test whether the stressor evoked a significant neuroendocrine stress response, we calculated repeated measures ANOVAs with group (stress group vs. stress-control group) as the independent variable and the 4 and 7 time points in which CA (NE and EPI) or cortisol were measured as repeated dependent variable.

To test whether catheter-insertion (intended to function as an open wound) would increase microbicidal potential of HMDM over time, we first calculated repeated measures ANCOVA in the stress-control group with the four WST-1 reduction time points (baseline, 1, 10, and 60 min after rest) as repeated dependent variable. Second, we tested whether the stress-control group significantly differed from the catheter-control group, again by means of ANCOVA with repeated WST-1 reduction as dependent variable and group as the independent variable while controlling for age and BMI as covariates. Third, to test for differences between the first and second WST-1 measurement in the catheter-control group we used paired Student’s *t*-tests.

In order to investigate stress reactivity of HMDM microbicidal potential in the stress study, we calculated repeated measures ANCOVA with group (stress group vs. stress-control group) as independent variable and the four WST-1 reduction time points as repeated dependent variable. Post-hoc testing of significant WST-1 reduction effects comprised (1) ANCOVAs to test for group differences in each measured WST-1 reduction time point, and (2) separate recalculation of repeated ANCOVAs in the stress group only.

To test for a potential mediation of microbicidal potential by stress hormone release, we calculated ANCOVAs to test whether NE, EPI or cortisol changes (i.e. ΔNE, ΔEPI, ΔCORT) mediate the stress effect on microbicidal activity of HMDM. According to Baron and Kenny [35] statistical mediation holds if (step 1) the independent variable (group, i.e. stress group vs. stress-control group) is associated with the supposed mediator (i.e. ΔNE, ΔEPI, ΔCORT), if (step 2) the independent variable (group) is associated with the dependent variable (repeated WST-1 reduction), and if (step 3) the mediator (i.e. ΔNE, ΔEPI, ΔCORT) is significantly associated with the dependent variable (repeated WST-1 reduction) while controlling for the independent variable (i.e. group) and the association between the independent variable (group) and the dependent variable (WST-1 reduction) is lower than in step 2 [35]. We favored ANCOVAs over multiple linear regression analyses to test for statistical mediation because steps 2 and 3 include repeatedly measured WST-1 reduction as the dependent variable. The use of multiple linear regression analysis would require artificial integration of the repeatedly measured dependent variable into a single measure index resulting in a loss of information.

Post-hoc testing of significant mediation effects comprised first, statistical evaluation of the indirect effect. To do this, we followed Preacher and Hayes [36] and performed non-parametric bootstrapping analyses with *n* = 5000 re-samples and 95% bias-corrected and accelerated (BCa) confidence intervals (CI). In these analyses, mediation is significant if zero is not included in the 95% BCa CI. Because bootstrapping analyses require single measure indices, AUCg of WST-1 reduction was entered as dependent variable. Second, we calculated partial correlations between significant endocrine mediator variables and each WST-1 reduction time point. Huynh-Feldt correction for repeated measures was applied where appropriate.

In *in vitro* experiments, differences between reference sample (without co-incubation) and samples exposed to NE in the presence or absence of phentolamine were tested with paired Student’s *t*-tests Analogous to testing HMDM stress reactivity, we calculated percentage changes in WST-1 reduction for each incubation condition in relation to reference sample as indicators of NE-induced changes in HMDM microbicidal potential.

Effect size parameters (*f*) were calculated from partial *η^2^*-values and are reported where appropriate (effect size conventions: *f*:.10 = small,.25 = medium,.40 = large).

## Results

### Characteristics of the Subject Groups

Table 1 provides the characteristics of the 24 stressed subjects and 17 stress-controls studied. The two groups did not differ significantly in the proportion of age, BMI, MAP, NE, and EPI levels. Cortisol baseline levels were higher in the stress group than in stress-control subjects. As expected, stressed subjects had higher stress hormone changes (ΔEPI; ΔNE; ΔCORT) than stress-controls. Due to technical problems, in two subjects of the stress-control group both NE data and EPI data were missing. In the catheter-control group, the mean age was 24±1.8 years and the mean BMI was 21±0.9 kg/m^2^. The three in-vitro study subjects were aged 25, 31, and 50 years. No further group characteristics were assessed.

**Table 1:** Characteristics of the 41 subjects studied.

	Stress group (*n* = 24)	Stress-control group (*n = *17)	*P*-ANOVA
Age (yr)	35.9±1.9 (20–50)	34.7±1.9 (22–49)	.659
BMI (kg/m^2^)	24.4±0.8 (18.7–36.0)	24.6±0.5 (22.5–30.1)	.872
MAP (mm Hg)	89.6±1.8 (75.8–105.5)	87.8±1.3 (80.8–102.5)	.454
NE baseline (pg/ml)	448.5±38.2 (115.3–841.7)	419.2±48.5 (166.2–765.6)	.638
EPI baseline (pg/ml)	20.8±3.1 (6.1–79.1)	20.9±3.5 (7.1–47.3)	.996
Cortisol baseline (nmol/l)	5.3±0.5 (2.9–13.7)	3.7±0.4 (1.6–8.2)	.034
ΔNE (pg/ml)	239.5±24.7 (25.6–511.0)	64.1±23.0(-109.5–212.9)	<.001
ΔEPI (pg/ml)	41.7±7.9 (5.3–157.5)	4.5±2.3 (-13.9–20.2)	.001
ΔCORT (nmol/l)	15.7±2.1 (-1.0–40.5)	0.7±0.8 (-1.6–12.3)	<.001

Values are given as means ± SEM (range). BMI, body mass index; MAP, mean arterial pressure; NE, norepinephrine; EPI, epinephrine; stress hormone changes (ΔNE, ΔEPI, ΔCORT) refer to the respective difference between the expected stress peak level minus baseline.

### Validation Check of the Neuroendocrine Stress Response

Stress induced significant increases in all three stress hormones as compared to stress-controls (NE: group×time interaction: *F*(3, 102) = 9.09, *p*<.001, *f = *.52; EPI: group×time interaction: *F*(2.3,77.6) = 10.18, *p*<.001, *f = *.55; cortisol: group×time interaction: *F*(2.8, 99.34) = 23.6, *p*<.001, *f = *.82; Figure 1A-C).

### Validation Check of the Catheter-insertion as Open Wound Paradigm

**Figure 2:**
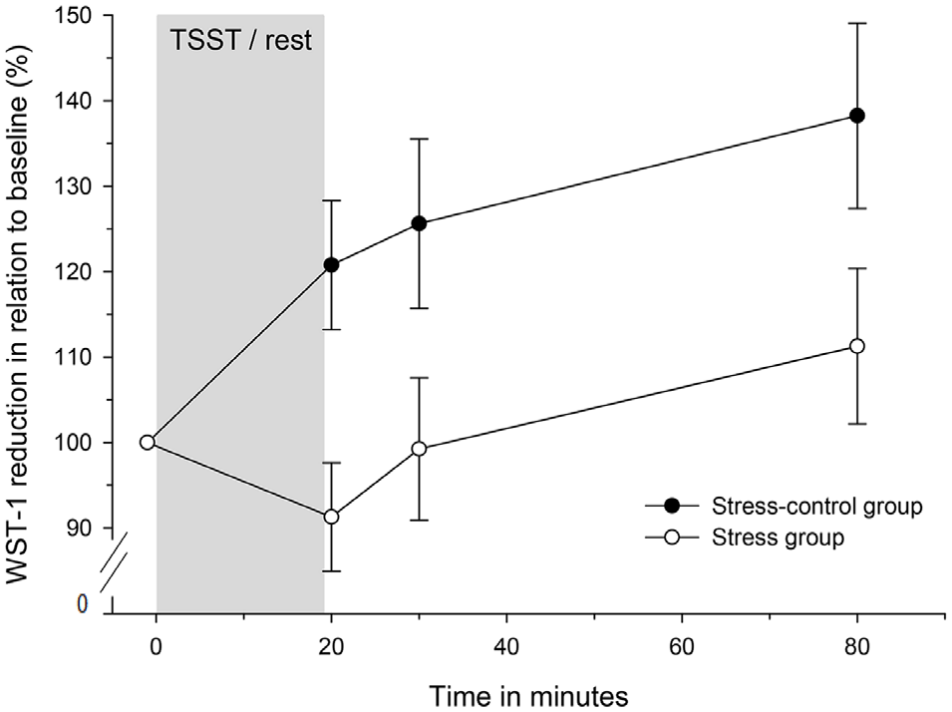
Course of WST-1 reduction over time in the stress and stress-control group. Values are given as means ± SEM.

As intended, the stress-control group displayed a significant increase in WST-1 reduction over time (*F*(3, 39) = 4.79, *p* = .006, *f = *.61, see Figure 2) and significantly differed from the catheter-control group (group (stress-control vs. catheter-control) ×time interaction: *F*(1, 21) = 11.52, *p* = .003, *f* = .74). In the catheter-control group WST-1 reduction scores were significantly lower in HMDM at the last assessment as compared to the first assessment (14,42±3.1%; *t*(5) = -4.75, *p* = .005). All subjects showed a decrease in WST-1 reduction from first to second assessment except one subject, who arrived at 10 a.m. (first assessment: 100%; second assessment: 102%).

### Stress Reactivity of WST-1 Reduction by Macrophages

Macrophage microbicidal potential in terms of WST-1 reduction was attenuated over time in the stress group as compared to the stress-control group (main effect of group (*F (*1, 36) = 8.06, *p* = .007, *f = *.47), group×time interaction (*F*(3, 108) = 2.24, *p* = .088; Figure 2). Post-hoc testing revealed that stressed subjects had a significantly reduced WST-1 reduction 1 min (*F*(1, 36) = 8.85, *p = *.005, *f = *.50) and 10 min (*F*(1, 36) = 4.1, *p = *.05, *f = *.33) after stress compared to stress-controls. The difference between groups was of borderline significance 60 min after TSST/rest (*F*(1, 36) = 3.6, *p = *0.066). In contrast to the stress-control group (see above), the stress group did not show a significant increase in WST-1 reduction over time (*F*(3, 60) = 0.50, *p = *.69).

### Mediation of WST-1 Reduction by Stress Hormone Changes

Statistical mediation testing revealed that the change in NE from baseline to 1 min after TSST/rest (ΔNE) significantly mediated the observed stress-induced attenuation of repeated macrophage microbicidal potential in terms of WST-1 reduction: (1) the group variable was significantly associated with ΔNE (*F*(1, 34) = 21.48, *p*<.001, *f = *.80) and (2) with repeated WST-1 reduction (*F(*1, 36) = 8.06, *p* = .007, *f = *.47). In addition, ΔNE was significantly associated with repeated WST-1 reduction (*F*(1, 33) = 7.7, *p = *.009, *f = *.48) while controlling for group, which lost its prior significance (*p = *.59). Neither EPI nor cortisol change coefficients were found to mediate repeated macrophage microbicidal potential, as both measures did not relate to repeated WST-1 reduction after controlling for group (*p’s* >.72). The post-hoc nonparametric bootstrapping analysis confirmed ΔNE as mediator between group and WST-1 reduction (BCa 95% CI: 4.14–44.72). Moreover, post-hoc correlation analyses showed that higher ΔNE levels were associated with lower WST-1 reduction scores 1 min (*r* = −.50, *p = *.002), 10 min (*r* = −.34, *p = *.044), and 60 min (*r* = −.56, *p*<.001) after TSST/rest. Table 2 depicts partial correlations between WST-1 reduction measures and stress hormone change coefficients.

### In vitro Experiments

**Figure 3:**
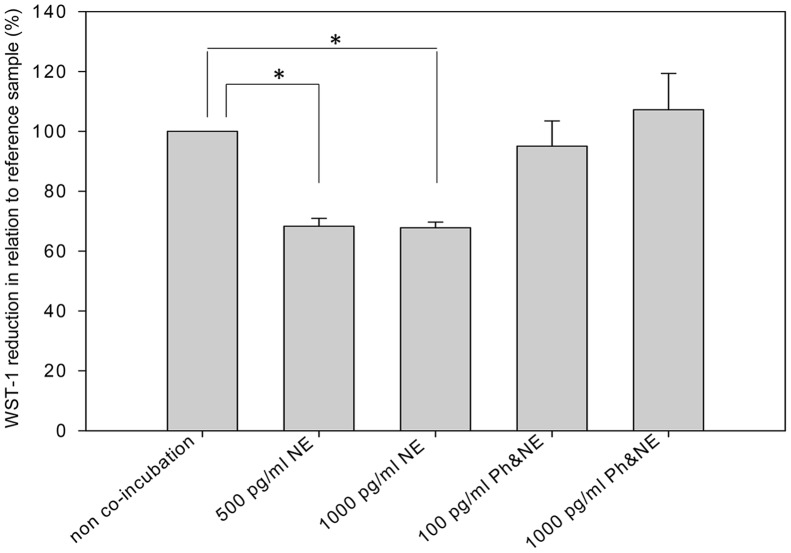
In vitro experiment with norepinephrine in the presence or absence of phentolamine. WST-1 reduction by *ex vivo* isolated human monocyte-derived macrophages after differential co-incubation. From the left: untreated cells (reference sample); co-incubation with 500 pg/ml norepinephrine; co-incubation with 1000 pg/ml norepinephrine; co-incubation with 100 pg/ml phenolamine before exposure to 500 pg/ml norepinephrine; co-incubation with 1000 pg/ml phenolamine before exposure to 500 pg/ml norepinephrine. Norepinephrine-treated monocyte-derived macrophages showed reduced superoxide anion production (*p*<.001). Alpha-adrenergic blockade with phentolamine before incubation with norepinphrine reversed this decrease. All *in vitro* experiments were repeated two times and carried out in cells of three female participants, rendering a total of six measurements. Values are given as mean ± SEM. NE, Norepinephrine; Ph, Phentolamine. *p<.001.

WST-1 reduction scores were significantly lower in HMDM incubated with both 500 and 1000 pg/ml NE than in HMDM without co-incubation (reference sample) (500 pg/ml NE: *t*(5) = -17.17, *p*<.001; 1000 pg/ml NE: *t*(5) = -17.51, *p*<.001, Figure 3). Adrenergic blockade with 100 pg/ml and 1000 pg/ml phentolamine before incubation with 500 pg/ml NE blocked the NE-induced decrease in WST-1 reduction (100 pg/ml phentolamine: *t*(5) = -0.59, *p* = .58; 1000 pg/ml phentolamine: *t*(5) = −.60, *p = *.58).

## Discussion

To our knowledge, this is the first study to investigate whether macrophage microbicidal potential is reduced by acute psychological stress after catheter insertion intended to induce an open wound and whether this stress effect is mediated by treatment (stress vs. rest)-induced stress hormone change. Additionally, we assessed pilot data to test whether catheter insertion as compared to non-permanent short-term cannula insertion would increase macrophage microbicidal potential during the testing period of our stress study. We used a WST-1 assay based on the reduction of WST-1 by superoxide anions produced by PMA-activated *ex vivo* isolated HMDM [24], [25], and measured plasma levels of the stress hormones NE, EPI, and cortisol. To validate our statistical mediation results we also performed *in vitro* experiments with NE in the presence or absence of phentolamine.

As hypothesized, we found an increase in WST-1 reduction of PMA-activated *ex vivo* isolated HMDM over time in the stress-control group but not in the stress group and not in the catheter-control group. WST-1 reduction by HMDM of the stressed subjects remained unchanged throughout the study period. Without insertion of a venous catheter, i.e. in the catheter-control group, we observed a decrease in WST-1 reduction by HMDM during the testing period. The stress-induced inhibition of stimulated macrophage microbicidal potential was statistically mediated by NE changes but did not significantly relate to EPI and cortisol changes. Higher NE stress responses (ΔNE) were associated with lower scores of WST-1 reduction by HMDM. In line with this, NE at post-stress levels reduced WST-1 reduction in our *in vitro* experiments. Furthermore, adrenergic blockade with phentolamine before incubation with NE completely blocked the observed decrease in WST-1 reduction. Taken together, our findings suggest that wound-induced stimulation of the microbicidal potential of HMDM is reduced by acute psychological stress, and that this effect of stress is mediated by stress-induced NE plasma levels. Notably, the effect of stress on WST-1 reduction was of medium to large or large effect size.

What are the potential implications of these findings and how do they relate to the literature? In contrast to our stressed subjects, we found that macrophage microbicidal potential of the stress-controls increased in terms of WST-1 reduction over time. We intentionally aimed for an increase in macrophage microbicidal potential in the stress-control group as we applied a standardized wound by simple insertion of a venous catheter more than two hours (165 min) prior to baseline assessment. After a review of the wound healing literature, we identified this time interval to be sufficiently long to allow initiation of wound healing processes and thus monocyte priming stimulation [6], [37]. In line with such reasoning, macrophage activity did not increase but even decrease in the catheter-control group during the monitoring period. Consequently, we interpret the observed continuous and apparently linear increase in microbicidal macrophage potential in the stress-control group as reflection of successful monocyte pre-activation due to wound healing initiation. Thus, our open wound paradigm seems to work in the expected direction. Notably, the observed decrease in WST-1 reduction over time in the catheter-control group might relate to circadian effects [14].

Our main finding is that microbicidal potential of HMDM of the stressed subjects remained unchanged throughout the study period. Psychological stress has been repeatedly shown to delay the process of wound healing [1], [2] in which macrophages are known to play a pivotal role [6]. Skin wound healing characteristically runs in consecutive and overlapping phases with the inflammatory phase as an early phase beginning within hours after wound application [37]. This phase aims at eliminating potential microbes, foreign particles and cell debris in the wound [6], [37]. After approximately 48 hours, the most prominent immune cells in the wound area are monocyte-derived macrophages, predominantly of the M1 macrophage phenotype [6], [37], [38]. These M1 macrophages are essential for successful completion of the inflammatory phase: They kill microbes by microbicidal activity and also promote inflammation by secreting pro-inflammatory cytokines, thus contributing to the progression to the next levels of wound healing [3], [8]. Notably, the differentiation method used in our study (i.e. co-incubation of *ex vivo* isolated monocytes with IFN-γ, TNF-α, and LPS) is known to result in M1 macrophages, i.e. our HMDM represent M1 macrophages [8]. After successful cleaning of the wound, macrophages appear to change their functional phenotype into M2 macrophages, which show increased effector functions important for the next phases of wound healing [39]. Given the importance of our HMDM as tissue M1 macrophages [6], [40]–[42], our findings suggest that the stress-induced delay in wound healing may be mediated by suppression of M1 macrophage microbicidal potential, the necessary precondition for microbicidal activity. Moreover, our cross-sectional analyses and *in vitro* experiments suggest that the stress-induced release of NE mediates the observed stress-induced attenuation of microbicidal macrophage potential. Interestingly, while the literature of stress effects on wound healing consistently demonstrates a stress-induced delay in wound healing [1], Dhabhar and colleagues postulate that acute stress contributes to immunoenhancement by both increased traffic of immune cells to target tissues soon after the beginning of stress and immune cell activation [43]. The reduced macrophage microbicidal potential that we observed after acute stress points to an immunosuppressive effect by acute stress. Thus, our findings appear at first glance to be more consistent with the findings from wound-healing studies than with those from Dhabhar et al. [43]. However, it cannot be ruled out that the stress-induced suppression of macrophage microbicidal potential is overcompensated by increased numbers of macrophages in target tissues as a consequence of acute stress. Moreover, it can also not be ruled out, that the suppressive effects of stress on macrophage microbicidal potential are adaptive if stress exposure persists for more than 10 min. Lastly, given that ROS can damage tissues once they reach a certain concentration [44], reduced macrophage activity (as a result of acute short-term stress) could represent a mechanism that protects target tissues from excessive and thus harmful concentrations of ROS. Considering this, our findings may extend the assumptions of Dhabhar et al. [43].

What mechanisms may underlie our observed findings? We propose the following hypothesized process to explain NE-mediated attenuation of wound-induced activation of HMDM microbicidal potential in the stress group as compared to the stress-control group: NE inhibits LPS-induced production of pro-inflammatory cytokines such as TNF-α [45], [46]. Given this ability of NE to inhibit stimulated cytokine release by monocytes, NE may inhibit monocyte/macrophage cytokine release (1) either during the *in vivo* wound-priming period when wound application-induced thrombin release may stimulate cytokine release and/or (2) during the *in vitro* differentiation period when LPS, TNF-α, and IFN-γ are used as stimulating agents. Consequently, NE may reduce cytokine concentrations and thus lower expression of NADPH oxidase subunits as prerequisites of PMA-induced NADPH oxidase activation [24]. Notably, NADPH oxidase is a membrane bound enzyme complex made up of several subunits and responsible for ROS formation in macrophages [47]. File S1 depicts the proposed process in detail. Notably, the observed stress-induced reduction of macrophage microbicidal potential was independent of chronic stress or cognitive stress appraisal (data not shown).

Some limitations of our study must be reported. First, we activated NADPH oxidase supraphysiologically using the synthetic stimulus PMA leading to increased NADPH oxidase activity in comparison to natural stimuli [24], [48]. We can only speculate as to whether stress reduces physiologically activated NADPH oxidase activity. Second, our mediation analysis is of a statistical nature. Although cross-sectional results clearly indicate mediation by NE, and inhibitory effects of NE on HMDM could be validated in our six in vitro experiments, further experimental studies are needed to verify this finding, particularly in larger sample sizes. Third, we cannot rule out that the FBS used in our WST-1 assay contains hydrocortisone. Thus, the non-significant mediation effect for cortisol on HMDM needs to be interpreted with care. A fourth limitation refers to the existing gender differences between participants of the stress study and the blood donators of the additional experiments (i.e. catheter-control group and *in vitro* subject group). While our main findings are restricted to healthy, medication-free, non-smoking men, the findings of the additional subsequent experiments were conducted in female subjects without applying any exclusion criteria. Notably, all female subjects seemed healthy although we did not verify this. Fifth, the sample sizes of the additional experiments are small. Given this, and given the limited gender selection (i.e. women only) results of the additional subsequently conducted experiments (i.e. catheter-control and *in vitro* data) should be considered as pilot or preliminary data. Further studies are needed to replicate our findings in other (and larger) samples and settings investigating both, men and women.

Our study does also have various strengths: It is the first human study to investigate stress reactivity of macrophage microbicidal potential after wound application. It combines both *in vivo* wound application and stress induction and *in vitro* measurement of M1 microbicidal potential. In addition to the fact that our study design included a resting stress-control group, a further strength was the standardized and simple wound application paradigm initiating wound healing processes in a natural manner. Furthermore, as monocytes/macrophages represent the dominating leukocyte subpopulation in the wound area for approximately 48 hours after wound application [6], [37], [38], our 48h-monocyte *in vitro* incubation period was intended to create maximum numbers of monocyte-derived M1 macrophages that possibly resemble *in vivo* macrophage activation processes at wound sites.

In sum, our findings suggest that microbicidal potential as the precondition for microbicidal activity of human M1 macrophages is inhibited by acute psychological stress. Furthermore, the mechanism by which stress decreases HMDM microbicidal potential may involve NE stress responses. Our findings might have implications for the stress-induced delay in wound healing. A better understanding of the mechanisms how psychological stress delays wound healing may provide important information for developing interdisciplinary intervention strategies.

## Supporting Information

File S1 Proposed process to explain stress-induced wound healing attenuation.
(DOC)
