# Supplementary material for: Acute Stress Reduces Wound-Induced Activation of Microbicidal Potential of Ex Vivo Isolated Human Monocyte-Derived Macrophages
Source: PLoS One. 2013 Feb 8;8(2):e55875. doi: 10.1371/journal.pone.0055875 (PMC3568075; doi:10.1371/journal.pone.0055875)
Supplement: File S1 — Proposed process to explain stress-induced wound healing attenuation. (DOC) [file pone.0055875.s001.doc]

**File S1.** **Proposed process to explain stress-induced wound healing attenuation.**

**
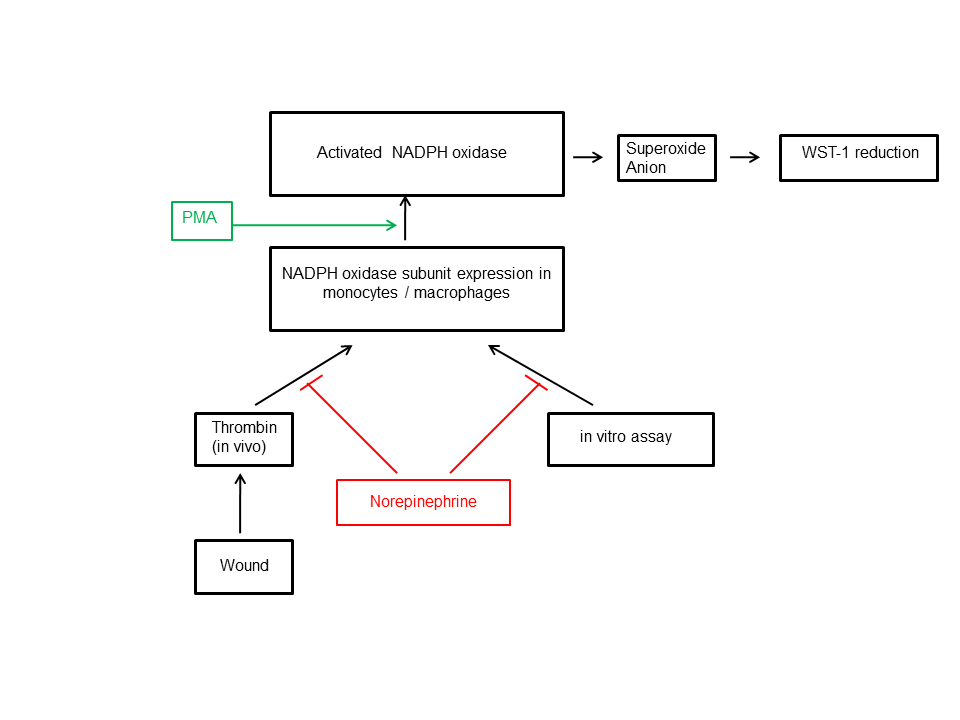
**

*Mechanism of superoxide anion production and subsequent WST-1 reduction.* An increase in superoxide anion^-^ induced WST-1 reduction results from the activation of the enzyme complex NADPH oxidase . Activation of NADPH oxidase is induced by phosphorylation of cytosolic subunits (hitherto identified subunits: p67phox, p47phox, p40phox, and RacGDP) and their subsequent translocation to membrane-bound subunits (hitherto identified: gp91phox p22phox) to form the complex . Notably, higher activation by a higher amount of NADPH oxidase complex formation results in higher superoxide anion reduction. An increase in NADPH oxidase complex formation is likely to result from increases in the number of NADPH oxidase subunits combined with phosphorylation of cytosolic subunits. Our *in vitro* assay comprises cytokine and LPS-induced stimulation of the expression of the NADPH oxidase subunits p22phox, p47phox, and p67phox, and subsequent activation with PMA, a substance that activates PKC, which in turn induces phosphorylation of cytosolic subunits . Thus, our 48h incubation procedure induces superoxide anion production in a standardized way.

*Our stress-control group showed an increase in superoxide anion induced WST-1 reduction over time.* Given that the four samples of a control person (obtained during an 80-min interval starting 165 min after catheter insertion) showed an increasing superoxide anion production over time, and given the standardized *in vitro* stimulation procedure, we assume that the catheter insertion (and thus the open wound application) may promote additional expression of NADPH oxidase subunits prior to the standardized *in vitro* stimulation procedure. Indeed, wound induction promotes thrombocytes to secrete the enzyme thrombin . Thrombin in turn stimulates monocytes to secrete inflammatory cytokines such as IFN-γ and TNF-α, which may further stimulate monocytes by autocrine and paracrine signal mediation . Interestingly, IFN-γ and TNF-α are both capable of stimulating expression of NADPH oxidase subunits . IFN-γ also increases expression of TLR 4, a well-known LPS-receptor . LPS (which is used as a stimulant in the subsequent standardized stimulation procedure) similarly increases NADPH oxidase subunit expression . In sum, this prior activation is assumed to result in increased NADPH oxidase subunit and LPS-receptor expression and may thus represent a kind of wound-induced priming of circulating monocytes as the precursors of M1 macrophages. We speculate that this hypothesized priming effect is responsible for the increase in superoxide anion production over time in our stress-control group.

*We observed lower WST-1 reduction in our stress group that is statistically mediated by NE increases*. NE has been repeatedly shown to inhibit LPS-induced production of pro-inflammatory cytokines such as TNF-α . Given the NE-induced inhibition of stimulated cytokine release by monocytes, NE may inhibit monocyte / macrophage cytokine release either during the *in vivo* wound priming period and / or during the *in vitro* differentiation period when LPS, TNF-α, and IFN-γ are used as stimulating agents. Consequently, NE may reduce cytokine concentrations and thus lower expression of NADPH oxidase subunits as prerequisites of NADPH oxidase activation.

**References**

1. Sakai M, Vonderheit A, Wei X, Kuttel C, Stemmer A (2009) A novel biofuel cell harvesting energy from activated human macrophages. Biosens Bioelectron 25: 68-75.

2. El-Benna J, Dang PM, Gougerot-Pocidalo MA, Elbim C (2005) Phagocyte NADPH oxidase: a multicomponent enzyme essential for host defenses. Arch Immunol Ther Exp (Warsz) 53: 199-206.

3. Bedard K, Krause KH (2007) The NOX family of ROS-generating NADPH oxidases: physiology and pathophysiology. Physiol Rev 87: 245-313.

4. He S, Blomback M, Bark N, Johnsson H, Wallen NH (2010) The direct thrombin inhibitors (argatroban, bivalirudin and lepirudin) and the indirect Xa-inhibitor (danaparoid) increase fibrin network porosity and thus facilitate fibrinolysis. Thromb Haemost 103: 1076-1084.

5. Mahdavian Delavary B, van der Veer WM, van Egmond M, Niessen FB, Beelen RH (2011) Macrophages in skin injury and repair. Immunobiology 216: 753-762.

6. Schroder K, Hertzog PJ, Ravasi T, Hume DA (2004) Interferon-gamma: an overview of signals, mechanisms and functions. J Leukoc Biol 75: 163-189.

7. Gauss KA, Nelson-Overton LK, Siemsen DW, Gao Y, DeLeo FR, et al. (2007) Role of NF-kappaB in transcriptional regulation of the phagocyte NADPH oxidase by tumor necrosis factor-alpha. J Leukoc Biol 82: 729-741.

8. DeLeo FR, Renee J, McCormick S, Nakamura M, Apicella M, et al. (1998) Neutrophils exposed to bacterial lipopolysaccharide upregulate NADPH oxidase assembly. J Clin Invest 101: 455-463.

9. van der Poll T, Jansen J, Endert E, Sauerwein HP, van Deventer SJ (1994) Noradrenaline inhibits lipopolysaccharide-induced tumor necrosis factor and interleukin 6 production in human whole blood. Infect Immun 62: 2046-2050.

10. Verhoeckx KC, Doornbos RP, van der Greef J, Witkamp RF, Rodenburg RJ (2005) Inhibitory effects of the beta-adrenergic receptor agonist zilpaterol on the LPS-induced production of TNF-alpha in vitro and in vivo. J Vet Pharmacol Ther 28: 531-537.
